# Friedel–Crafts acylation of benzene derivatives in tunable aryl alkyl ionic liquids (TAAILs)

**DOI:** 10.3762/bjoc.19.20

**Published:** 2023-02-23

**Authors:** Swantje Lerch, Stefan Fritsch, Thomas Strassner

**Affiliations:** 1 Professur für Physikalische Organische Chemie, Technische Universität Dresden, 01062 Dresden, Germany

**Keywords:** Friedel–Crafts acylation, homogeneous catalysis, ionic liquids, iron catalysis, TAAILs

## Abstract

An iron(III) chloride hexahydrate-catalyzed Friedel–Crafts acylation of benzene derivatives in tunable aryl alkyl ionic liquids (TAAILs) has been developed. Through optimization of the metal salt, reaction conditions and ionic liquids, we were able to design a robust catalyst system that tolerates different electron-rich substrates under ambient atmosphere and allows for a multigram scale.

## Introduction

The Friedel–Crafts acylation is one of the oldest metal-catalyzed reactions in organic chemistry [1] and allows for the synthesis of a broad range of diverse compounds [2–5]. Starting from electron-rich aromatic compounds, acylation is possible by an organic acid chloride/acid anhydride and a Lewis acid [6–7]. In the course of the development of ionic liquids (ILs) as a reaction medium for chemical reactions [8–9], the Friedel–Crafts reaction was also examined [10–16]. First protocols were presented by Wilke and colleagues in 1986 [17]. At that time, chloroaluminate ionic liquids were used both as a solvent and catalyst [18–19], but these systems proved to be unstable under ambient air conditions and prone to decompose in the presence of water [20].

In the following years, ionic liquids proved to be a remarkable class of compounds due to their high thermal and chemical stability, their negligible vapor pressure and high versatility in terms of chemical structure and usage [21–24].

Successive research for the Friedel–Crafts acylation lead to the development of various reaction protocols using different metal salts [25–30] and ionic liquids [31–35] to acylate [36–41], alkylate [42–44], benzylate [45–47] and alkenylate [48–49] different benzene derivatives. But surprisingly, a robust protocol using commercially available and cost-efficient metal salts in a water and air stable ionic liquid is still hard to find [50–51].

In this contribution, we present the use of imidazolium-based tunable aryl alkyl ionic liquids (TAAILs) in a catalytic Friedel–Crafts acylation. This relatively new class of ionic liquids [52] previously proved to be a potent reaction medium in catalytic hydrosilylation [53], hydroamination and hydroarylation [54], as well as for the synthesis of nanoparticles [55–56].

## Results and Discussion

**Scheme 1:**
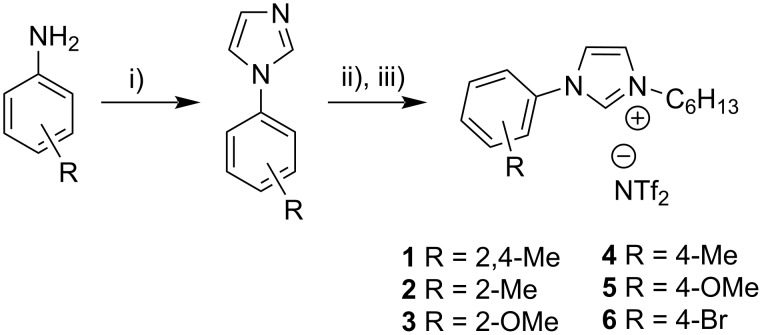
Synthesis of TAAILs. i) 1 equiv glyoxal, 2.1 equiv formaldehyde, 2 equiv NH_4_Cl, MeOH, 65 °C, ii) 1.1 equiv C_6_H_13_Br, THF, 70 °C, iii) 1.1 equiv LiNTf_2_, DCM/MeOH/H_2_O, rt.

Ionic liquids **1**–**6** were synthesized via a three-step procedure starting from commercially available aniline derivatives (see Scheme 1). First, the arylimidazole is obtained through a ring closing reaction using an aniline derivative, glyoxal, formaldehyde and ammonium chloride. The following alkylation with hexyl bromide yields the bromido ionic liquid. TAAILs **1**–**6** are then formed by an anion exchange reaction using lithium bis(trifluoromethylsulfonyl)imide (LiNTf_2_). All reactions are generally tolerant towards different aryl substitutions, substitution patterns, alkyl chain lengths and can be carried out in a multigram scale [57].

**Scheme 2:**
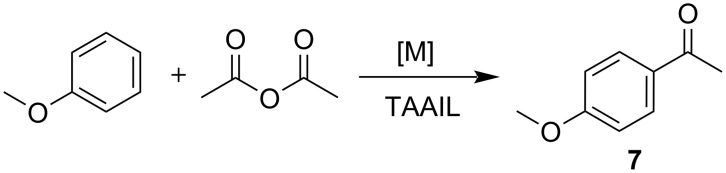
Model reaction for the Friedel–Crafts acylation.

The acylation of the electron-rich benzene derivative anisole with acetic anhydride (Ac_2_O) to acetanisole **7** was chosen as the model reaction (Scheme 2) [36].

As different metal salts are known to be effective catalysts for this reaction [58–60], several metal chlorides were tested for their capability to catalyze the acylation in TAAILs. To our surprise, neither water-free AlCl_3_ nor the hexahydrate were able to catalyze the reaction in TAAILs. The hydrates of several rare-earth metal chlorides (CeCl_3_, NdCl_3_ and SmCl_3_) were used as well, but only small amounts of product (less than 5%) were observed, whereas the hydrates of cobalt and iron chloride were able to catalyze the acylation successfully (Table 1). The best results were achieved with the FeCl_3_ hexahydrate. Interestingly, we also used the anhydrous salt for this reaction, it did, however, show inferior performance compared to the hydrate. No formation of the *ortho*-isomer of acetanisole **7** was observed while screening the different Lewis acids, the reaction is regioselective for the *para*-position.

**Table 1:** Yields achieved with TAAILs **1**–**6** and different catalysts^a^.

TAAIL	CoCl_2_·6H_2_O	FeCl_3_·6H_2_O	FeCl_3_

**1**	28%	60%	53%
**2**	27%	61%	49%
**3**	17%	47%	47%
**4**	33%	60%	52%
**5**	30%	61%	50%
**6**	43%	62%	60%

^a^Reaction conditions: 1 mmol anisole, 1.3 equiv Ac_2_O, 10 mol % catalyst, *T* = 60 °C, *t* = 24 h, 0.5 g TAAIL.

**Figure 1:**
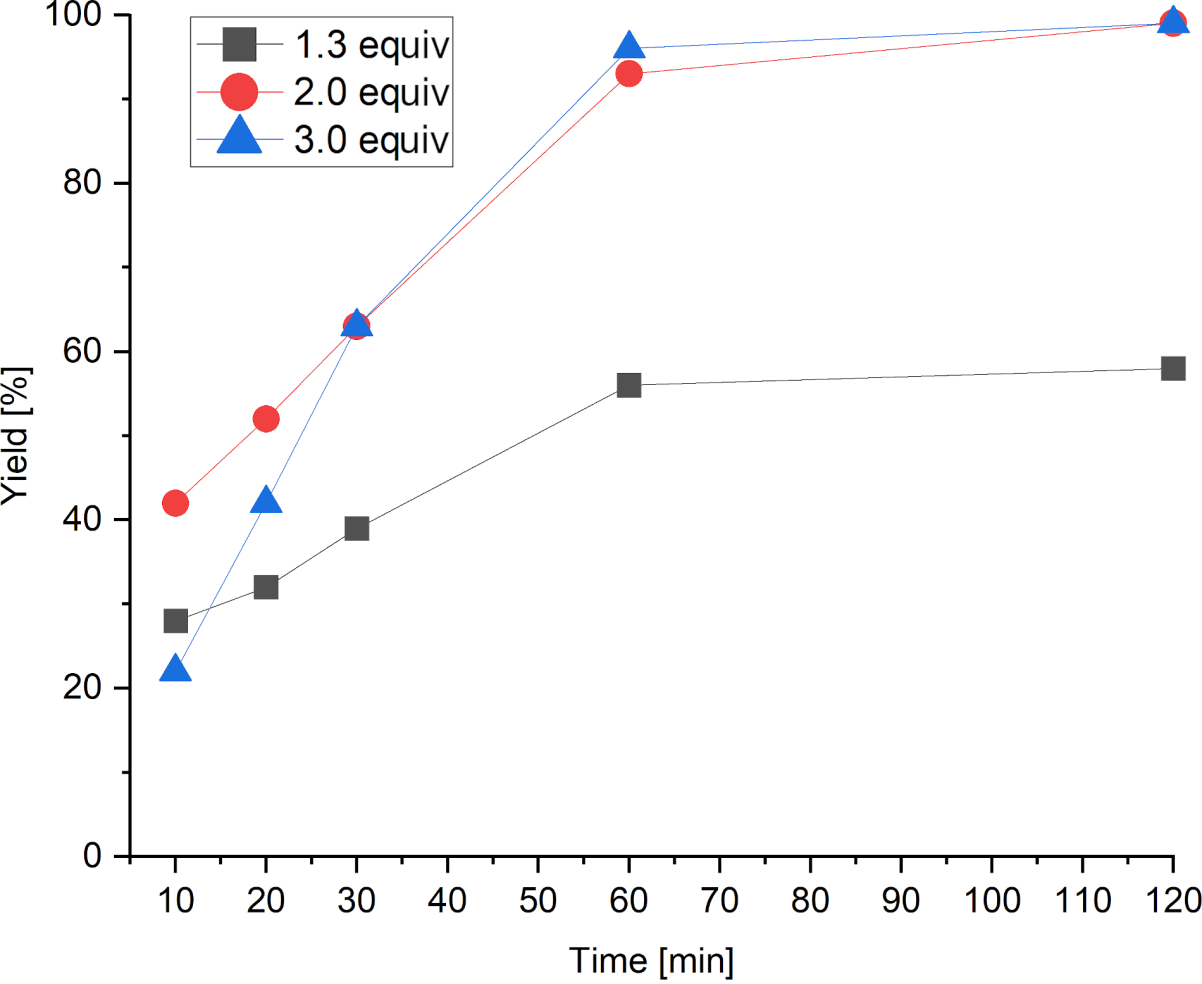
Time-dependent analysis of the reaction using varying amounts of anhydride. Reaction conditions: 1 mmol anisole, 1.3–3.0 equiv Ac_2_O, 10 mol % FeCl_3_·6H_2_O, *T* = 60 °C, 0.5 g TAAIL **6**.

TAAIL **6** was chosen for further optimizations because it appeared to be a slightly better reaction medium compared to the other TAAILs. To optimize the yield of the reaction and to reduce the reaction time, different amounts of acetic anhydride were used and a time-dependent investigation of the catalytic system was performed. Samples of the reaction mixture were taken after 10, 20, 30, 60 and 120 minutes and analyzed via GC–MS. The results are shown in Figure 1.

The results show that full conversion of the starting material is achieved after two hours when using 2 or 3 equivalents of anhydride. Since the use of more than 2 equivalents of anhydride does not appear to be beneficial for the reaction, further investigations were done using 2 equivalents of the anhydride. To see how temperature and catalyst load impact the yield of the reaction, the reaction was carried out at 40 °C while the catalyst load was varied between 2 and 10 mol %. The results are given in Table 2.

**Table 2:** Variation of catalyst load and reaction temperature^a^.

Entry	FeCl_3_·6H_2_O (mol %)	*T* (°C)	Yield (%)

1	10	60	97
2	5	60	87
3	2	60	65
4	10	40	82
5	5	40	68
6	2	40	51

^a^Reaction conditions: 1 mmol anisole, 2 equiv Ac_2_O, *t* = 2 h, 0.5 g TAAIL **6**.

As can be expected, the lower temperature results in a reduced yield after 2 hours of reaction time. Decreasing the catalyst load also leads to diminished yields, but the loss is rather small compared to the reduced catalyst concentration. To further characterize our catalytic system, the acylation was performed on a larger scale to investigate whether upscaling changes the performance of the catalytic system. Table 3 shows the results of the reactions and their respective turn over numbers (TONs) [61].

**Table 3:** Upscaling experiments^a^.

Entry	Scale (mmol)	FeCl_3_·6H_2_O (mol %)	Yield (%)	TON^b^

1	1	10	99	10
2	2	10	94	9
3	2	5	86	17
4	4	10	90	9
5	4	2.5	65	26
6	6	10	92	9
7	6	1.7	58	34

^a^Reaction conditions: 1 mmol anisole, 2 equiv Ac_2_O, 0.5 g TAAIL **6**, *T* = 60 °C, *t* = 2 h, ^b^TON = turn over number (amount of product/ amount of catalyst).

10 mol % of catalyst was used for Table 3, entries 1, 2, 4 and 6. Both the yield and the calculated TONs decrease only slightly when increasing the scale of the reaction. For Table 3, entries 3, 5 and 7, 0.1 mmol catalyst were used which corresponds to 5 mol % at the 2 mmol scale and 1.7 mol % at 6 mmol scale. Even though the yields decrease with smaller catalyst loadings, the TONs increase considerably.

**Scheme 3:**
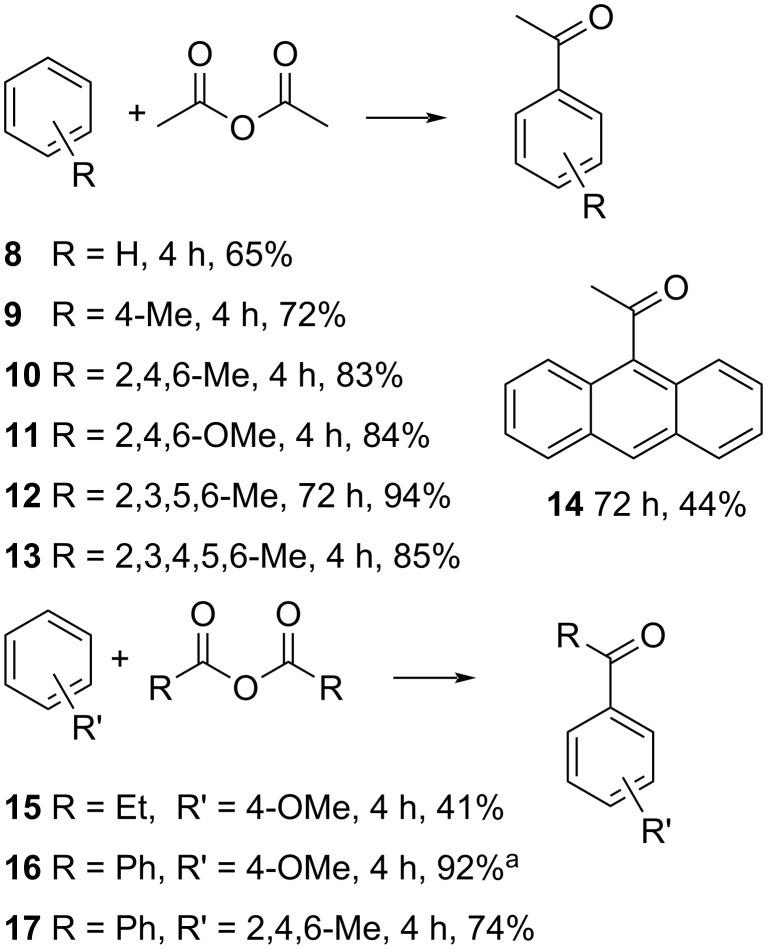
Scope of the Friedel–Crafts acylation. Reaction conditions: 1 mmol benzene derivative, 2 equiv anhydride, 10 mol % FeCl_3_·6H_2_O, *T* = 60 °C, 0.5 g TAAIL **6**; ^a^5% of the *ortho*-isomer were formed.

We also tested the scope of the reaction by using various substrates with our catalytic system, since both the benzene derivative and the anhydride can be varied (Scheme 3).

Different benzene derivatives were successfully used in the acylation with acetic anhydride with yields between 65% and 94% (**8**–**13**). The reaction time varied between four and 72 hours and the reaction was stopped after full conversion of the benzene derivative (detected via GC–MS). The products were isolated via flash column chromatography to determine the yield. Anthracene was selectively acylated at the 9-position (**14**) using acetic anhydride. The use of other anhydrides was also tested: propionic anhydride and benzoic anhydride lead to yields between 41% and 92%, with minor formation of the *ortho*-isomer when using benzoic acid anhydride. Bromobenzene, which is electron poor and less reactive towards acylation, was also used as a substrate in the acylation reaction, but no product was observed. This indicates that the ionic liquid **6**, having the same substituent, does not undergo acylation during the catalysis. Finally, we compared our catalytic system with commercially available imidazolium and phosphonium-based ILs (Table 4).

**Table 4:** Comparison with other ILs^a^.

Entry	Ionic liquid	Yield (%)

1	[EMIm]NTf_2_	77
2	[BMIm]NTf_2_	96
3	[BBMIm]NTf_2_	86
4	[P66614]NTf_2_	81
5	[P66614]Cl	0

^a^Reaction conditions: 1 mmol anisole, 2 equiv Ac_2_O, 10 mol % FeCl_3_·6H_2_O, *T* = 40 °C, *t* = 4 h, 0.5 g IL.

The imidazolium-based IL [EMIm]NTf_2_ converts anisole with a yield of 77% (see Table 4, entry 1), whereas [BMIm]NTf_2_ shows a similar performance as TAAIL **6**. Using IL 1,3-dibutyl-2-methylimidazolium NTf_2_ (Table, entry 3), carrying no hydrogen atom at the C2 position of the imidazolium ring, 86% of product were obtained. The phosphonium-based IL [P66614]NTf_2_ again shows an inferior performance compared to TAAIL **6**. Upon changing the anion of the ionic liquid, catalytic activity is suppressed and product formation is not observed at all (Table 4, entry 5). An additional advantage of ILs is the reusability of the catalytic system, we were able to reuse our system for three consecutive runs.

## Conclusion

In conclusion, we have established a robust system for a catalytic Friedel–Crafts acylation. The reaction was carried out at moderate temperatures of 40 °C to 60 °C under air, was scalable to gram-scale and tolerated different electron-rich benzene derivatives as well as anhydrides. The catalytic performance depends on the choice of ionic liquid and the TAAILs outperform commercially available ILs in our model reaction.

## Supporting Information

File 1 Experimental procedures, characterization data, copies of ^1^H and ^13^C NMR spectra.
