# Supplementary material for: Friedel–Crafts acylation of benzene derivatives in tunable aryl alkyl ionic liquids (TAAILs)
Source: Beilstein J Org Chem. 2023 Feb 23;19:212–6. doi: 10.3762/bjoc.19.20 (PMC9972882; doi:10.3762/bjoc.19.20)
Supplement: File 1 — Experimental procedures, characterization data, copies of 1H and 13C NMR spectra. [file Beilstein_J_Org_Chem-19-212-s001.pdf]

BEILSTEIN JOURNAL OF ORGANIC CHEMISTRY

Supporting Information

for

Friedel–Crafts acylation of benzene derivatives in tunable aryl alkyl ionic liquids (TAAILs)

Swantje Lerch, Stefan Fritsch and Thomas Strassner

Beilstein J. Org. Chem. **2023,** *19,* 212–216. doi:10.3762/bjoc.19.20

Experimental procedures, characterization data, copies of ^{1}H and ^{13}C NMR spectra

License and Terms: This is a supporting information file under the terms of the Creative Commons Attribution License (https://creativecommons.org/licenses/by/4.0). Please note that the reuse, redistribution and reproduction in particular requires that the author(s) and source are credited and that individual graphics may be subject to special legal provisions.
The license is subject to the *Beilstein Journal of Organic Chemistry* terms and conditions: (https://www.beilstein-journals.org/bjoc/terms)

Contents

General remarks	S1
Catalytic procedure of the Friedel–Crafts acylation in TAAILs	S1
Products of the Friedel–Crafts acylation	S2–S7
NMR-Spectra	S8–S18
References	S19–S20

General remarks

All chemicals were obtained from common suppliers and used without further purification. Dichloromethane was distilled prior to use, other solvents were used as received. ^1H and ^{13}C- NMR-spectra were acquired on a Bruker Avance 300, Bruker DRX 500 and Bruker Avance 600 spectrometers. ^1H and ^{13}C spectra were referenced internally using the solvent resonances (^1H: 7.26 ppm, ^{13}C: 77.0 ppm for $CDCl_3$). Chemical shifts are given in ppm, coupling constants *J* in Hz. Elemental analyses were performed by the microanalytical laboratory of our institute on a Hekatech EA 3000 Euro Vector elemental analyser.

Synthetic procedures as well as analytical data of the six substituted aryl imidazoles, the bromido ionic liquids and the bis(trifluoromethanesulfonyl)imide (NTf_2) ionic liquids have been previously described by our group.[1]

Catalytic procedure of the Friedel–Crafts acylation in TAAILs

All catalytic reactions were performed on a 1 mmol scale. The ionic liquid, the metal salt, the anhydride and the benzene derivative were placed in a 10 ml crimp vial with a magnetic stirring bar inside and the vial was closed with a butyl rubber septum. The mixture was placed in an aluminium block and the mixture was stirred at the given temperature for the indicated amount of time. After cooling to room temperature, dodecane (130 mg) was added as an internal standard and the mixture was diluted with 5 ml dichloromethane and stirred at room temperature for five minutes. An aliquot of 0.2 ml was placed onto a plug of silica and rinsed with 10 ml of dichloromethane. The resulting solution was analysed via GC–MS. All yields are given as an average of two runs.

For the determination of the isolated yield, the reaction mixture was diluted with DCM and adsorbed onto silica gel. The product was isolated via flash column chromatography.

Products of the Friedel–Crafts acylation

4-Methoxy-acetophenone (**7**).[2]

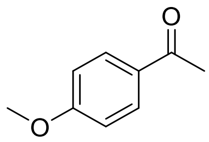

Product obtained as a yellow oil (124 mg, 0.82 mmol, 82%) after column chromatography (isohexane:ethyl acetate 6:1).

^{1}H-NMR ($CDCl_3$, 300 MHz): δ (ppm) 7.90 - 8.02 (m, 2 H, H_{ar}), 6.90 – 7.03 (m, 2 H, H_{ar}), 3.87 (s, 3 H, $OC\mathit{H}_3$), 2.55 (s, 3 H, $C\mathit{H}_3$).

^{13}C-NMR ($CDCl_3$, 75 MHz): δ (ppm) 196.8 (*C*O), 163.5 ($\mathit{C}_{ar}O$), 130.5 (C_{ar}), 113.6 ($C_{ar}H$), 55.4 ($O\mathit{C}H_3$), 26.3 ($\mathit{C}H_3$).

Elemental analysis: calc. for: $C_9H_{10}O_2$: C: 71.98 %, H: 6.71 %,
found: C: 71.93 %, H: 7.02 %.

Acetophenone (**8**).[3]

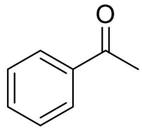

Product obtained as a colorless oil (78 mg, 0.65 mmol, 65%) after column chromatography (isohexane/ethyl acetate 8:2).

^{1}H-NMR ($CDCl_3$, 300 MHz): δ (ppm) 7.93 - 7.99 (m, 2 H, H_{ar}), 7.53 - 7.61 (m, 1 H, H_{ar}), 7.41 - 7.50 (m, 2 H, H_{ar}), 2.61 (s, 3 H, $C\mathit{H}_3$).

^{13}C-NMR ($CDCl_3$, 75 MHz): δ (ppm) 198.1 (*C*O), 137.1 ($\mathit{C}_{ar}H$), 133.1 ($\mathit{C}_{ar}H$), 128.5 ($\mathit{C}_{ar}H$), 128.3 ($\mathit{C}_{ar}H$), 26.6 ($\mathit{C}H_3$).

Elemental analysis: calc. for: $C_8H_8O \cdot 0.15\ H_2O$: C: 78.21%, H: 6.81%,
found: C: 77.88%, H: 6.50%.

4'-Methyl-acetophenone (**9**).[4]

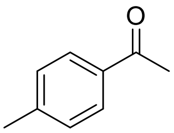

Product obtained as a colorless oil (97 mg, 0.72 mmol, 72%) after column chromatography (isohexane/ethyl acetate 8:2).

^{1}H-NMR ($CDCl_3$, 300 MHz): δ (ppm) 7.86 (d, *J* = 8.25 Hz, 2 H, H_{ar}), 7.22 - 7.29 (m, 2 H, H_{ar}), 2.58 (s, 3 H, CH_3), 2.41 (s, 3 H, CH_3).

^{13}C-NMR ($CDCl_3$, 75 MHz): δ (ppm) 197.8 (*C*O), 143.8 (C_{ar}), 134.7 (C_{ar}), 129.2 (C_{ar}H), 128.4 (C_{ar}H), 26.5 (*C*H_3), 21.6 (*C*H_3).

Elemental analysis: calc. for: $C_9H_{10}O \cdot 0.12\ H_2O$:		C: 79.29%, H: 7.57%,
	found:	C: 78.97%, H: 7.27%.

2', 4', 6'-Trimethylacetophenone (**10**).[5]

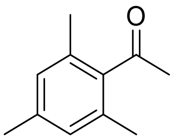

Product obtained as a pale yellow oil (129 mg, 0.83 mmol, 83%) after column chromatography (ethyl acetate:isohexane 1:10).

^{1}H-NMR ($CDCl_3$, 300 MHz): δ (ppm) 6.85 (d, *J* = 0.57 Hz, 2 H, H_{ar}), 2.47 (s, 3 H, CH_3), 2.29 (s, 3 H, CH_3), 2.23 (s, 6 H, CH_3).

^{13}C-NMR ($CDCl_3$, 75 MHz): δ (ppm) 208.6 (*C*O), 139.9 (C_{ar}), 138.3 (C_{ar}), 132.3 (C_{ar}), 128.5 (*C*H), 32.2 (*C*H_3), 21.0 (*C*H_3), 19.1 (*C*H_3).

Elemental analysis:	calc. for: $C_{11}H_{14}O$:	C: 81.44 %, H: 8.70 %,
	found:	C: 81.61 %, H: 8.88 %.

2,4,6-Trimethoxyacetophenone (**11**).[6]

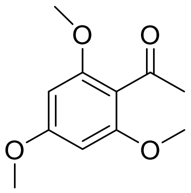

Product obtained as a pale yellow solid (176 mg, 0.84 mmol, 84%) after column chromatography (isohexane:ethyl acetate, 10:1).

^{1}H-NMR ($CDCl_3$, 300 MHz): δ (ppm) 6.11 (s, 2 H, H_{ar}), 3.83 (s, 3 H, OCH_3), 3.80 (s, 6 H, OCH_3), 2.46 (s, 3 H, CH_3).

^{13}C-NMR ($CDCl_3$, 75 MHz): δ (ppm) 201.7 (*C*O), 162.3 (C_{ar}), 158.3 (C_{ar}), 113.7 (C_{ar}), 90.6 (*C*H), 55.8 (OCH_3), 55.4 (OCH_3), 32.5 (CH_3).

Elemental analysis:	calc. for: $C_{11}H_{14}O_4$:	C: 62.85 %, H: 6.71 %,
	found:	C: 62.52 %, H: 6.69 %.

2,3,5,6-Tetramethylacetophenone (**12**).[7]

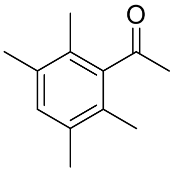

Product obtained as a pale yellow solid (165 mg, 0.94 mmol, 94%) after column chromatography (isohexane:ethyl acetate, 10:1).

^{1}H-NMR ($CDCl_3$, 300 MHz): δ (ppm) 6.96 (s, 1 H, H_{ar}), 2.47 (s, 3 H, CH_3), 2.22 (s, 6 H, CH_3), 2.10 (s, 6 H, CH_3).

^{13}C-NMR ($CDCl_3$, 75 MHz): δ (ppm) 209.8 (*C*O), 143.0 (C_{ar}), 134.4 (C_{ar}), 131.5 (*C*H), 127.6 (C_{ar}), 32.8 (CH_3), 19.4 (CH_3), 15.9 (CH_3).

Elemental analysis:	calc. for: $C_{12}H_{16}O$:	C: 81.77 %, H: 9.15 %,
	found:	C: 81.45 %, H: 9.38 %.

2,3,4,5,6-Pentamethylacetophenone (**13**).[8]

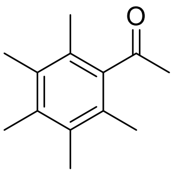

Product was obtained as a pale brown solid (154 mg, 0.85 mmol, 85%) after column chromatography (isohexane/ethyl acetate, 10:1).

^{1}H-NMR ($CDCl_3$, 300 MHz): δ (ppm) 2.47 (s, 3 H, C*H*$_3$), 2.25 (s, 3 H, C*H*$_3$), 2.20 (s, 6 H, C*H*$_3$), 2.15 (s, 6 H, C*H*$_3$).

^{13}C-NMR ($CDCl_3$, 75 MHz): δ (ppm) 210.1 (*C*O), 140.9 (*C*$_{ar}$), 135.3 (*C*$_{ar}$), 133.1 (*C*$_{ar}$), 127.0 (*C*$_{ar}$), 33.1 (*C*H$_3$), 17.1 (*C*H$_3$), 16.6 (*C*H$_3$), 15.9 (*C*H$_3$).

Elemental analysis:	calc. for $C_{13}H_{18}O$:	C: 82.06 %, H: 9.53 %;
	found:	C: 81.94 %, H: 9.89 %.

9-Acetylanthracene (**14**).[9]

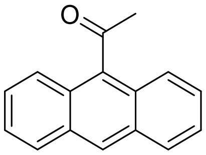

Product was obtained as a light brown solid (96 mg, 0.44 mmol, 44%) after column chromatography (isohexane/ethyl acetate, 10:1).

^{1}H-NMR ($CDCl_3$, 300 MHz): δ (ppm) 8.50 (s, 1 H, *H*$_{ar}$), 8.02 – 8.09 (m, 2 H, *H*$_{ar}$), 7.83 – 7.90 (m, 2 H, *H*$_{ar}$), 7.47 – 7.58 (m, 4 H, *H*$_{ar}$), 2.83 (s, 3 H, C*H*$_3$).

^{13}C-NMR ($CDCl_3$, 75 MHz): δ (ppm) 208.1 (*C*O), 136.7 (*C*$_{ar}$), 131.1 (*C*$_{ar}$), 128.8 (*C*$_{ar}$), 128.2 (*C*$_{ar}$), 126.8 (*C*$_{ar}$), 126.6 (*C*$_{ar}$), 125.5 (*C*$_{ar}$), 124.3 (*C*$_{ar}$), 33.9 (*C*H$_3$).

Elemental analysis:	calc. for $C_{16}H_{12}O$:	C: 87.25 %, H: 5.49 %,
	found:	C: 86.94 %, H: 5.73 %.

para-Methoxypropiophenone (**15**).[10]

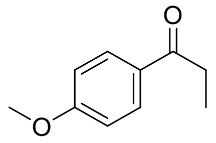

Product was obtained as a yellow oil (68 mg, 0.41 mmol, 41%) after column chromatography (isohexane:ethyl acetate, 10:1).

^{1}H-NMR ($CDCl_3$, 300 MHz): δ (ppm) 7.92 – 7.99 (m, 2 H, H_{ar}), 6.91 – 6.97 (m, 2 H, H_{ar}), 3.88 (s, 3 H, OCH_3), 2.96 (q, J=7.24 Hz, 2 H, CH_2), 1.22 (t, J=7.27 Hz, 3 H, CH_3).

^{13}C-NMR ($CDCl_3$, 75 MHz): δ (ppm) 199.5 (CO), 163.3 (C_{ar}), 130.2 (C_{ar}), 130.0 (C_{ar}), 113.6 (C_{ar}), 55.4 (OCH$_3$), 31.4 (CH$_2$), 8.4 (CH$_3$).

Elemental analysis:	calc. for $C_{10}H_{12}O_2$:	C: 73.15 %, H: 7.37 %,
	found:	C: 72.91 %, H: 7.45 %.

4-Methoxybenzophenone (**16**).[11]

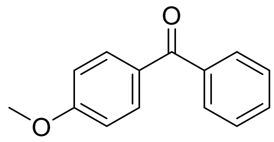

Product was obtained as a pale red oil (195 mg, 0.92 mmol, 92%) after column chromatography (isohexane:ethyl acetate, 10:1).

^{1}H-NMR ($CDCl_3$, 300 MHz): δ (ppm) 7.82 – 7.87 (m, 2 H, H_{ar}), 7.74 – 7.80 (m, 2 H, H_{ar}), 7.45 – 7.58 (m, 3 H, H_{ar}), 6.95 – 7.01 (m, 2 H, H_{ar}), 3.90 (s, 3 H, OCH_3).

^{13}C-NMR ($CDCl_3$, 75 MHz): δ (ppm) 195.6 (CO), 163.2 (C_{ar}), 138.3 (C_{ar}), 132.6 (C_{ar}), 131.9 (C_{ar}), 130.1 (C_{ar}), 129.7 (C_{ar}), 128.2 (C_{ar}), 113.5 (C_{ar}), 55.5 (OCH$_3$).

Elemental analysis:	calc. for $C_{14}H_{12}O_2$:	C: 79.23 %, H: 5.70 %,
	found:	C: 79.06 %, H: 5.83 %.

2,4,6-Trimethylbenzophenone (**17**).[12]

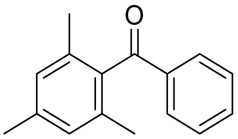

Product was obtained as a yellow oil (74 mg, 0.74 mmol, 74%) after column chromatography (isohexane:ethyl acetate, 10:1).

^{1}H-NMR ($CDCl_3$, 300 MHz): δ (ppm) 7.76 – 7.88 (m, 2 H, H_{ar}), 7.54 – 7.64 (m, 1 H, H_{ar}), 7.40 – 7.50 (m, 2 H, H_{ar}), 6.91 (d, J=0.57 Hz, 2 H, H_{ar}), 2.34 (s, 3 H, CH_3), 2.09 (s, 6 H, CH_3).

^{13}C-NMR ($CDCl_3$, 75 MHz): δ (ppm) 200.8 (*C*O), 138.5 (C_{ar}), 137.3 (C_{ar}), 136.9 (C_{ar}), 134.2 (C_{ar}), 133.5 (C_{ar}), 129.4 (C_{ar}), 128.8 (C_{ar}), 128.3 (C_{ar}), 21.1 (*C*H_3), 19.3 (*C*H_3).

Elemental analysis:	calc. for $C_{16}H_{16}O$:	C: 85.68 %, H: 7.19 %,
	found:	C: 85.86 %, H: 7.33 %.

NMR Spectra

^{1}H-NMR compound **7**

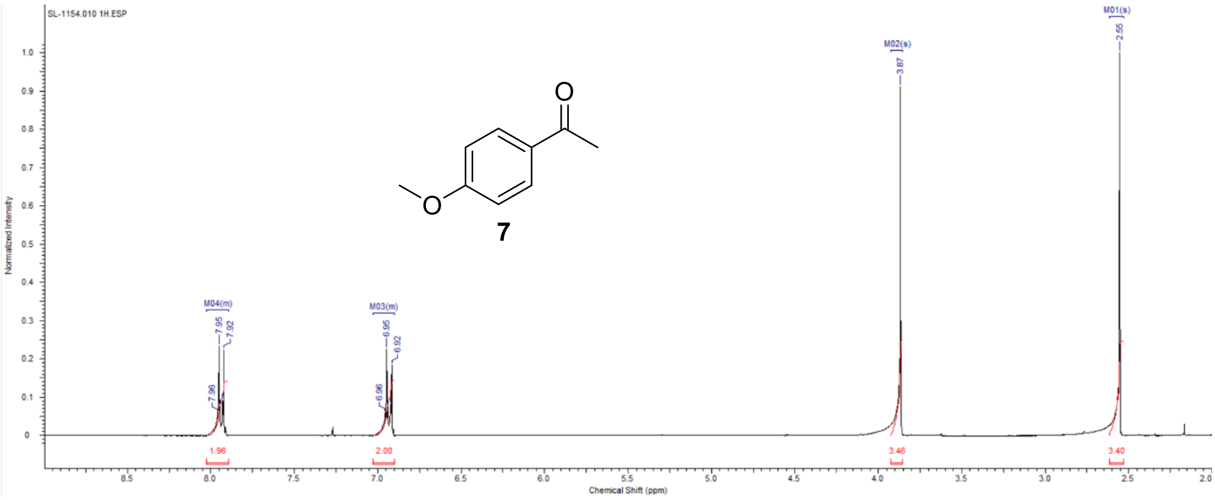

^{13}C-NMR compound **7**

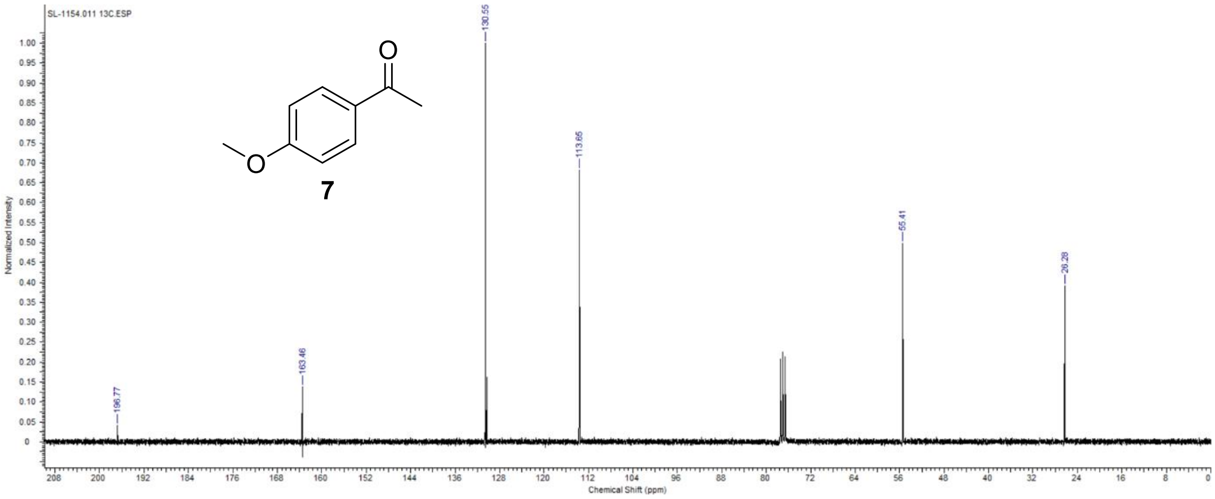

^{1}H-NMR compound **8**

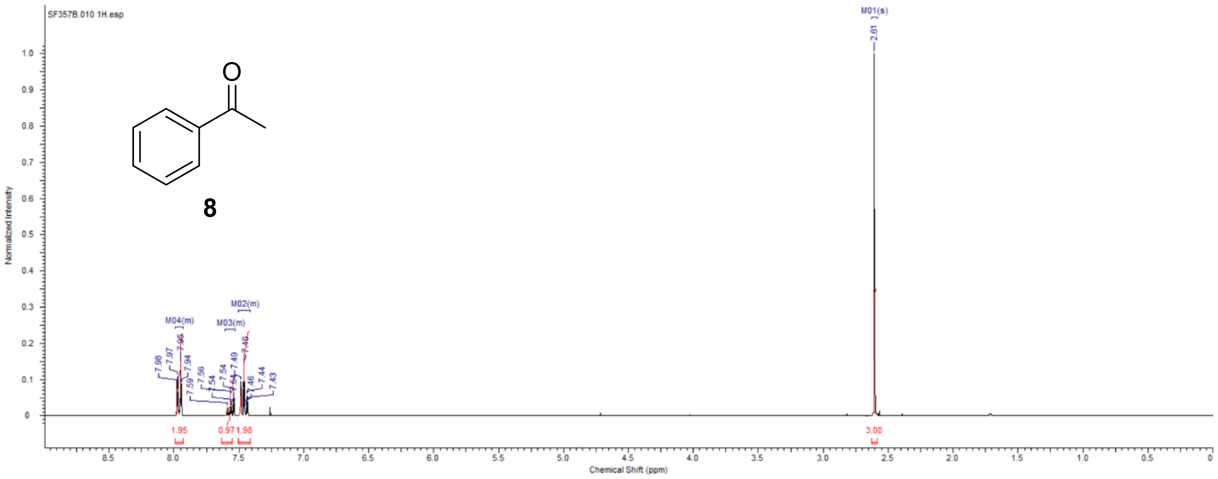

^{13}C-NMR compound **8**

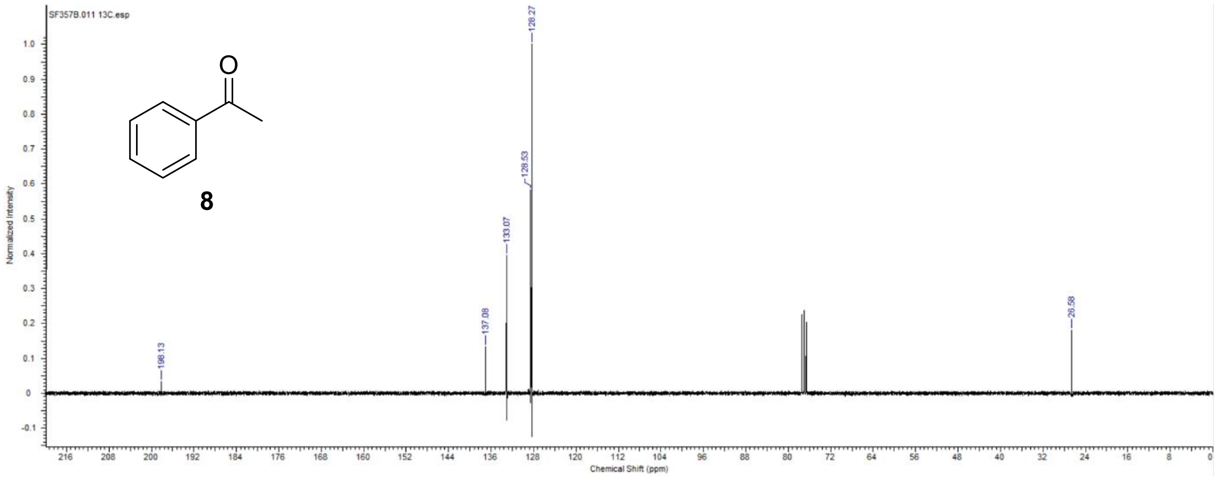

^{1}H-NMR compound **9**

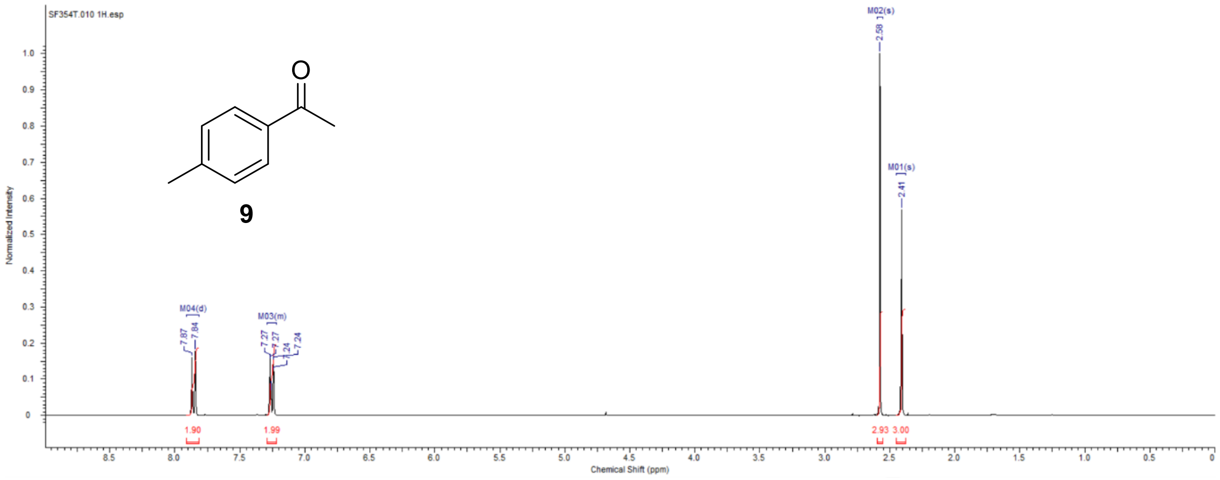

^{13}C-NMR compound **9**

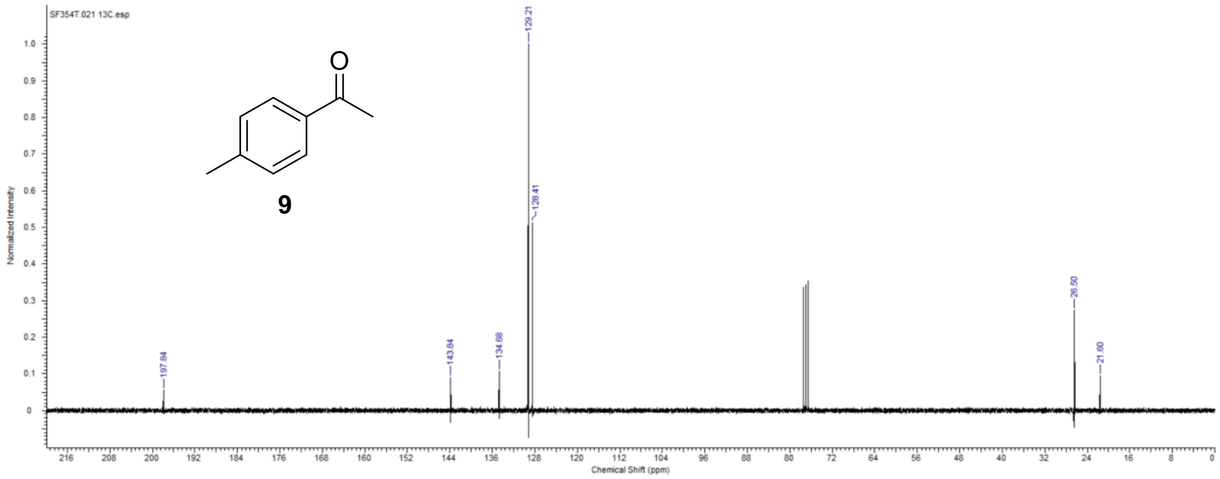

^{1}H-NMR compound **10**

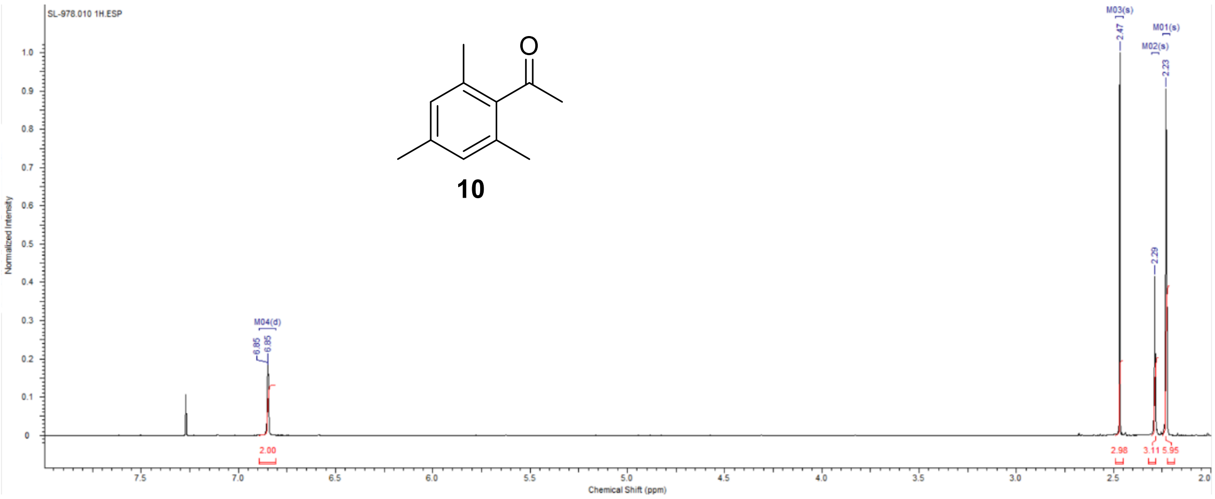

^{13}C-NMR compound **10**

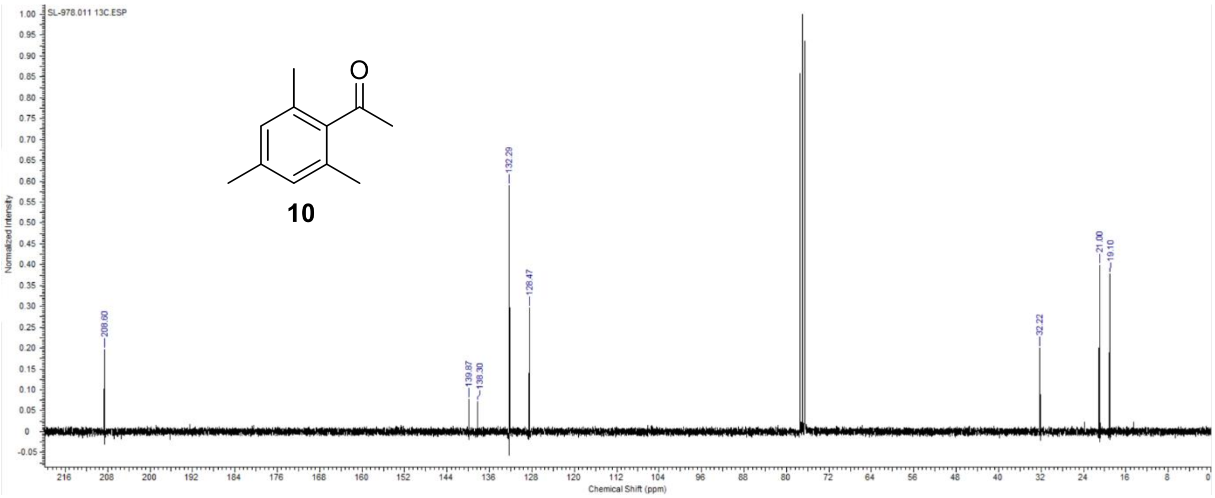

^{1}H-NMR compound **11**

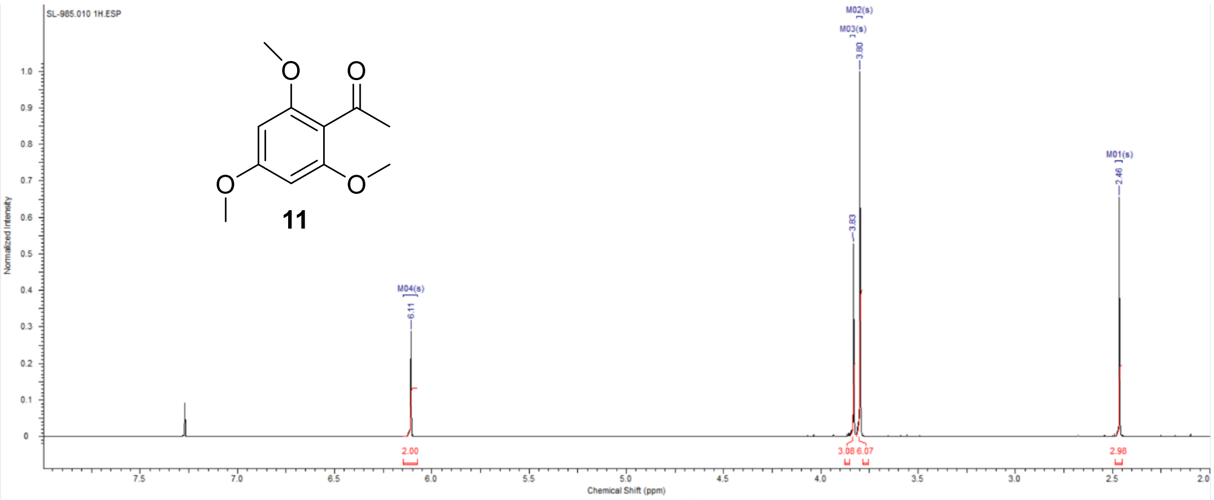

^{13}C-NMR compound **11**

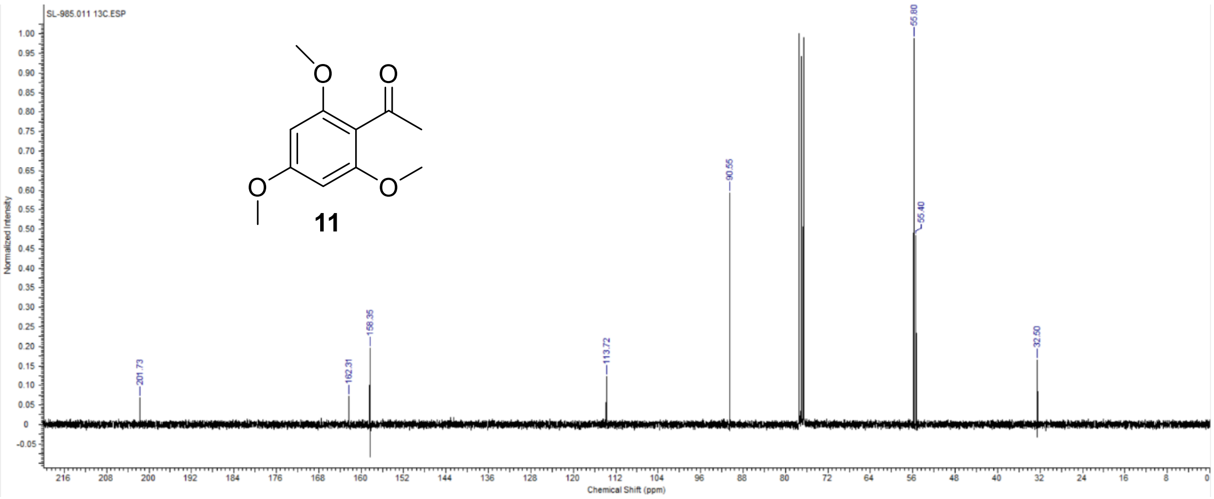

^{1}H-NMR compound **12**

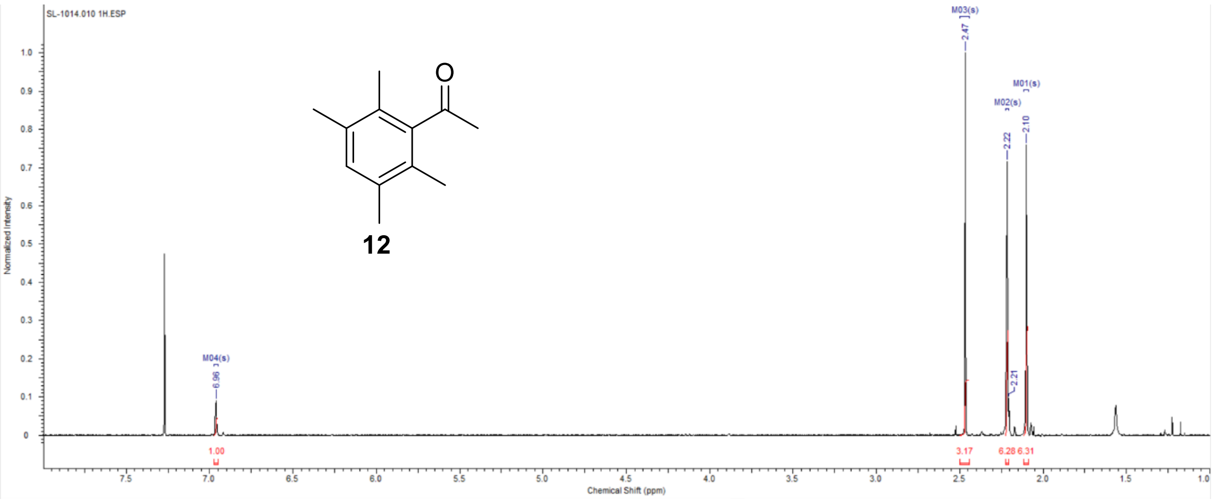

^{13}C-NMR compound **12**

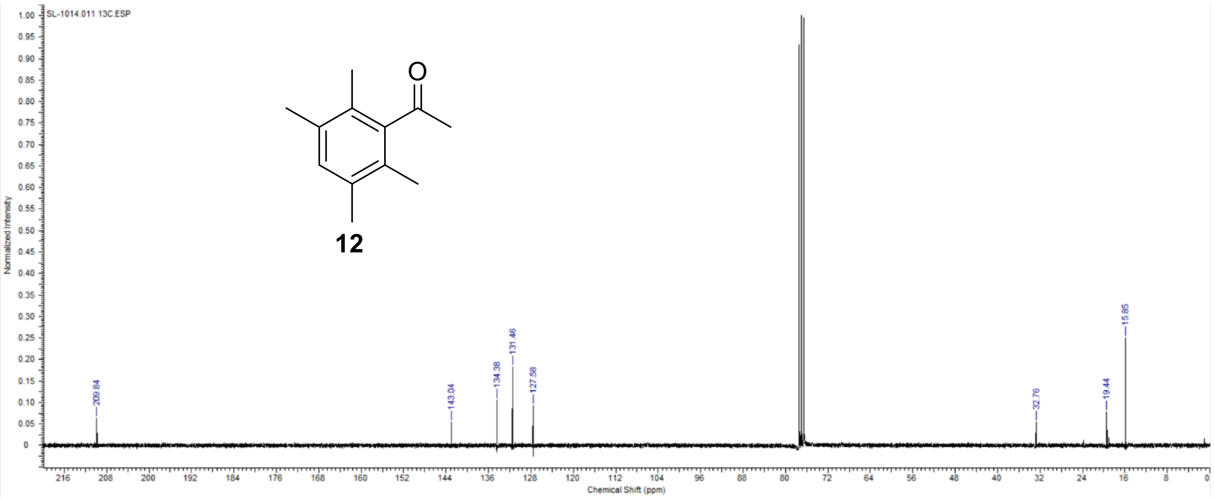

^{1}H-NMR compound **13**

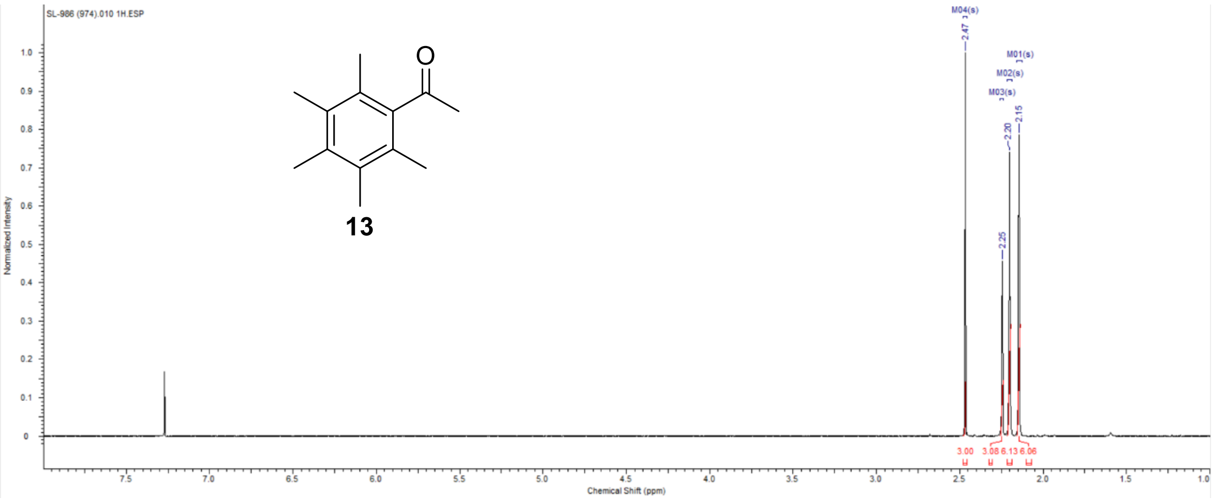

^{13}C-NMR compound **13**

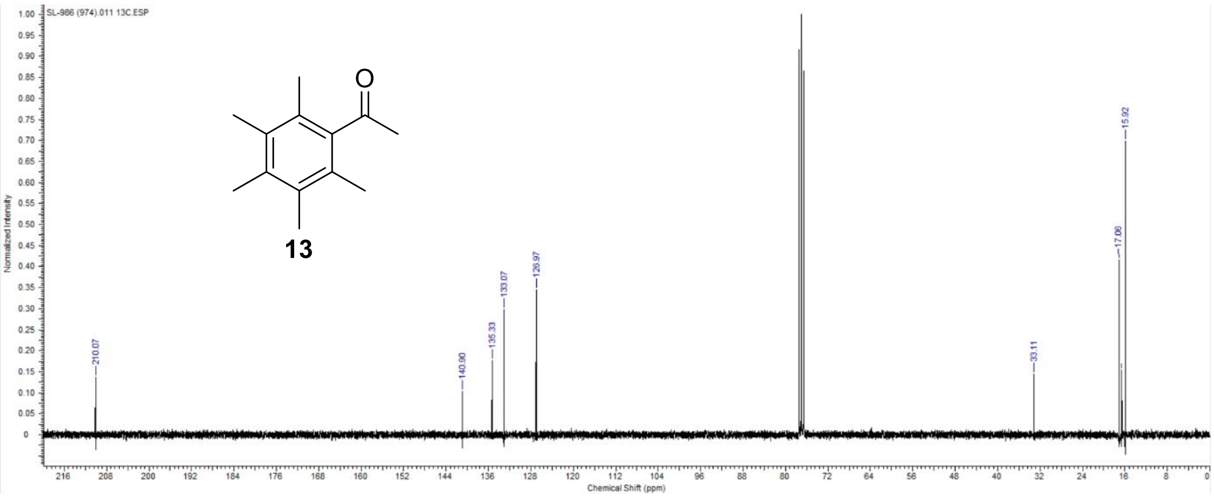

^{1}H-NMR compound **14**

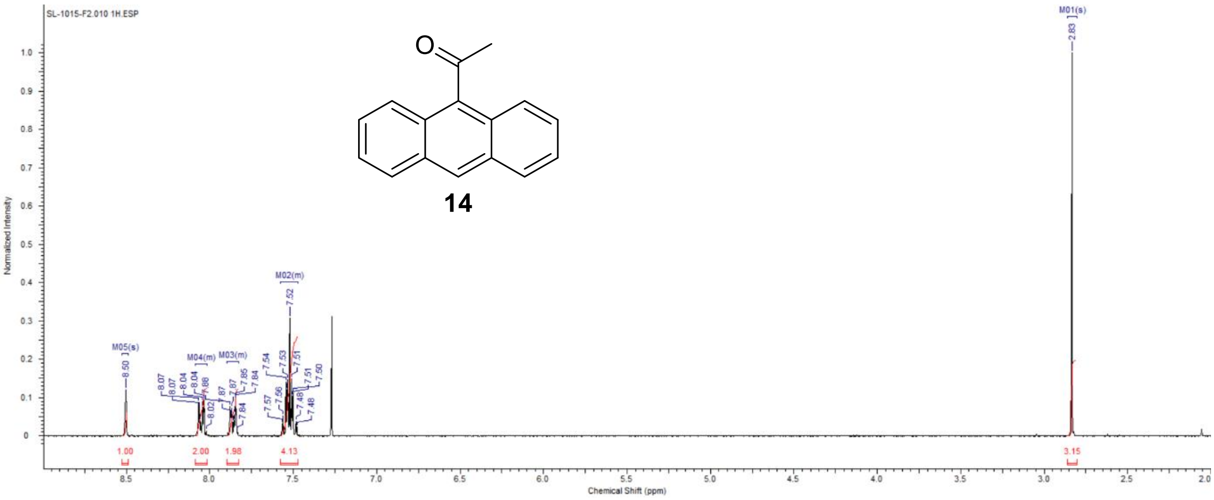

^{13}C-NMR compound **14**

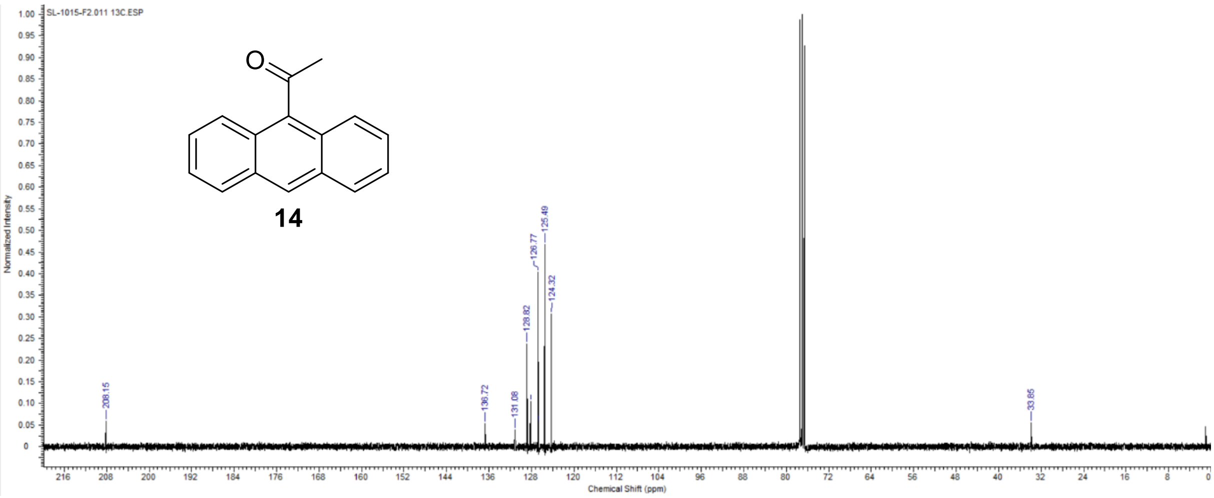

^{1}H-NMR compound **15**

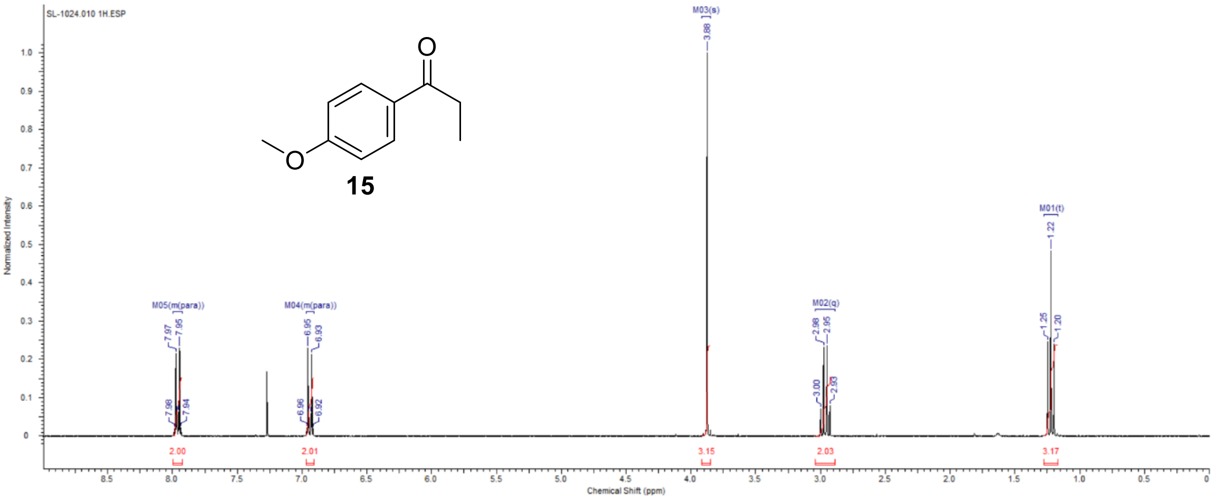

^{13}C-NMR compound **15**

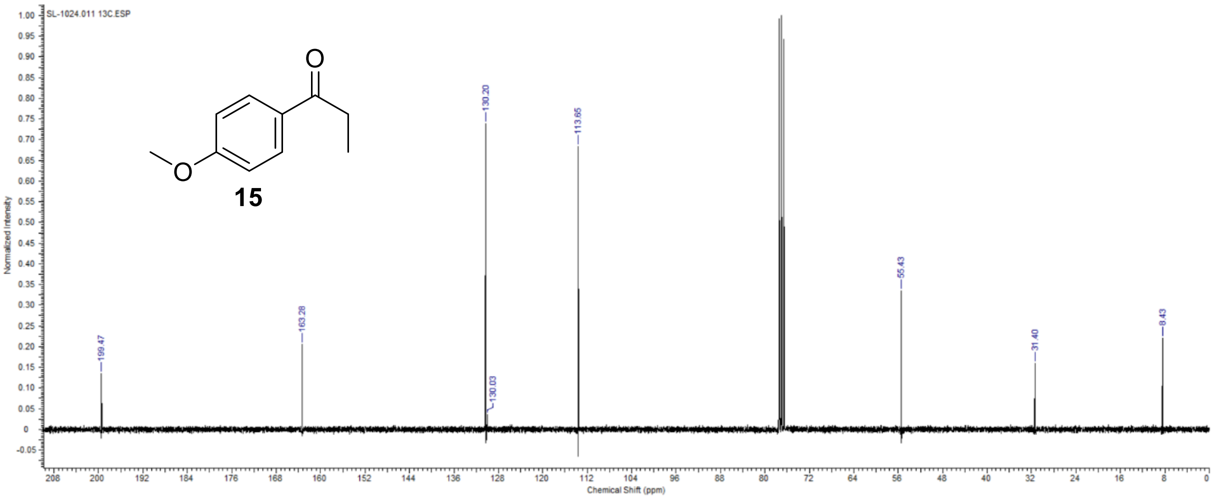

^{1}H-NMR compound **16**

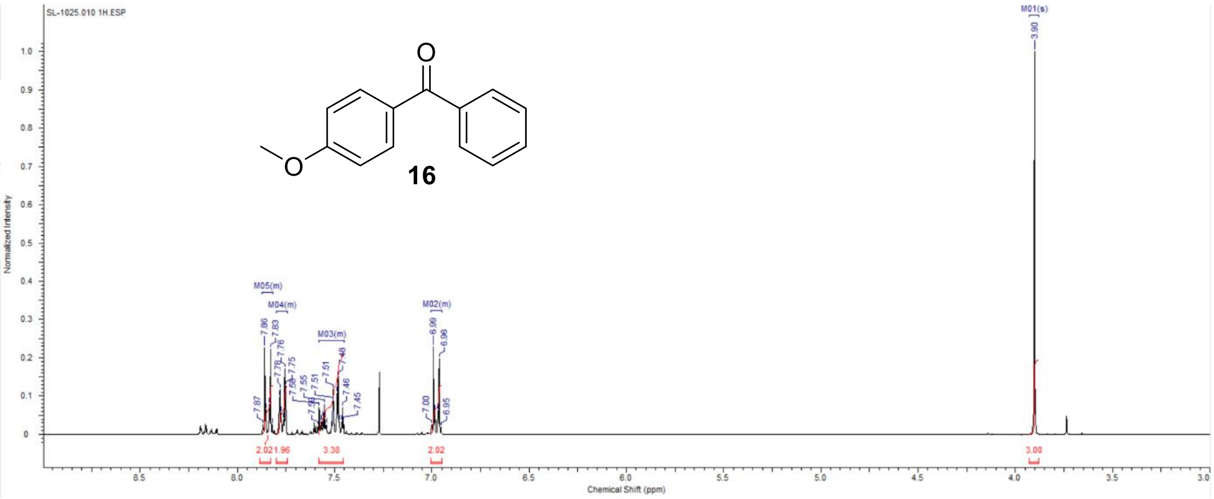

^{13}C-NMR compound **16**

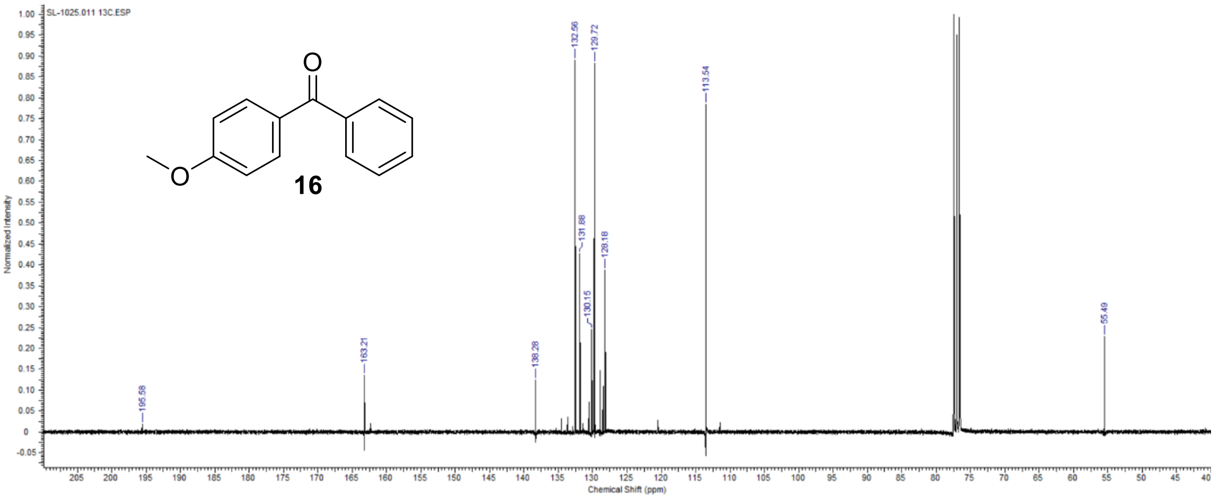

^{1}H-NMR compound **17**

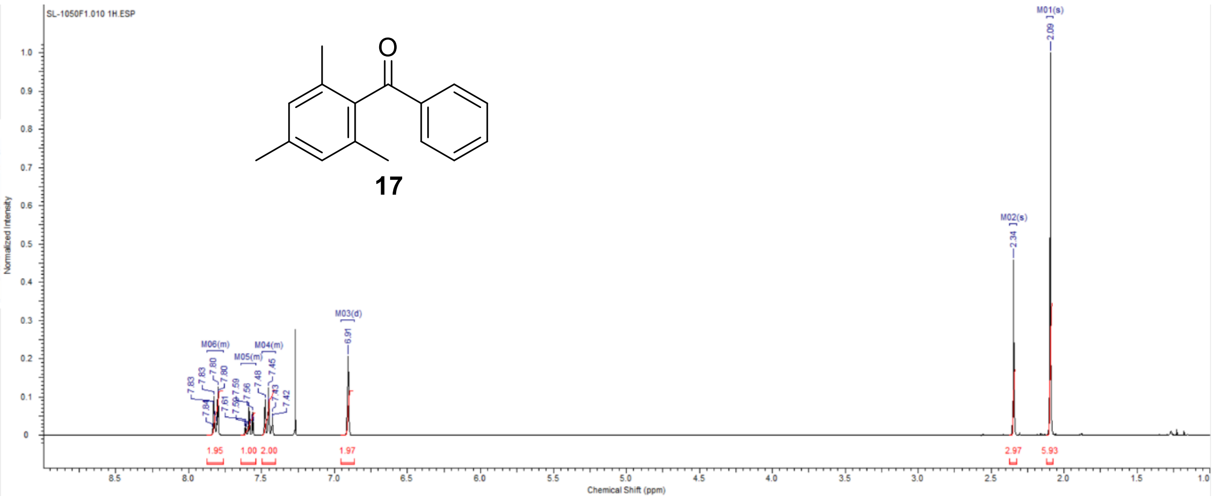

^{13}C-NMR compound **17**

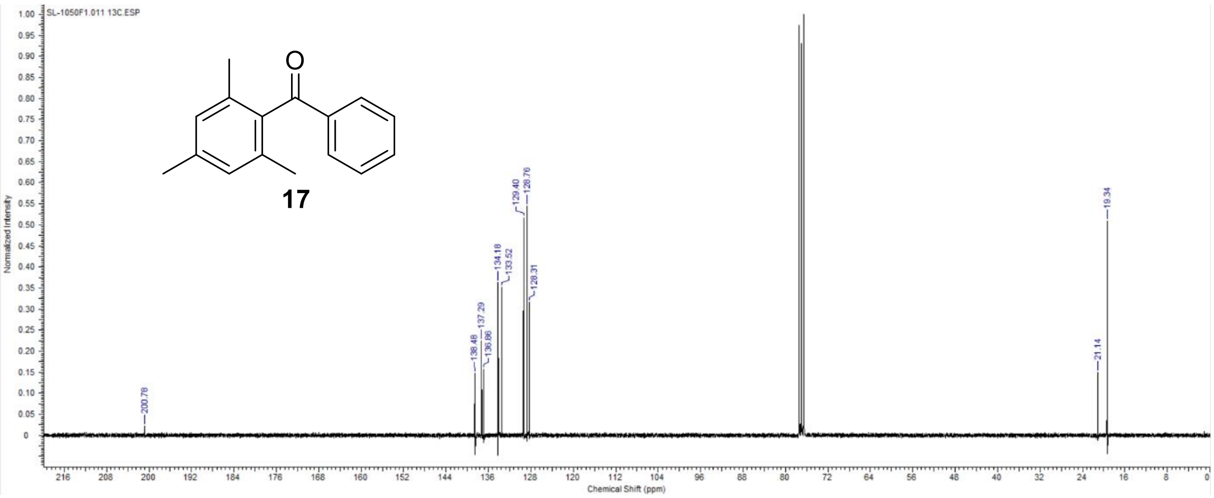

References

1. Lerch, S.; Strassner, T. Synthesis and Physical Properties of Tunable Aryl Alkyl Ionic Liquids (TAAILs). *Chem. Eur. J.* **2021**, 27, 15554-15557.

2. Yuan, Y.; Shi, X.; Liu, W. Transition-Metal-Free, Chemoselective Aerobic Oxidations of Sulfides and Alcohols with Potassium Nitrate and Pyridinium Tribromide or Bromine. *Synlett* **2011**, 4, 559-564.

3. Bhattacharjya, A.; Klumphu, P.; Lipshutz, B. H. Ligand-free, palladium-catalyzed dihydrogen generation from TMDS: dehalogenation of aryl halides on water. *Org. Lett.* **2015,** 17, 1122–1125.

4. Mo, J.; Xu, L.; Xiao, J. Ionic liquid-promoted, highly regioselective Heck arylation of electron-rich olefins by aryl halides. *J. Am. Chem. Soc.* **2005,** 127, 751–760.

5. Skillinghaug, B.; Sköld, C.; Rydfjord, J.; Svensson, F.; Behrends, M.; Sävmarker, J.; Sjöberg, P. J. R.; Larhed, M. Palladium(II)-catalyzed desulfitative synthesis of aryl ketones from sodium arylsulfinates and nitriles: scope, limitations, and mechanistic studies. *J. Org. Chem.* **2014**, 79, 12018-12032.

6. Zhang, B.; Duan, D.; Ge, C.; Yao, J.; Liu, Y.; Li, X.; Fang, J. Synthesis of xanthohumol analogues and discovery of potent thioredoxin reductase inhibitor as potential anticancer agent. *J. Med. Chem.* **2015**, 58, 1795-1805.

7. Budesínský, M.; Kulhánek, J.; Böhm, S.; Cigler, P.; Exner, O. ^{13}C and ^{1}H nuclear magnetic resonance of methyl-substituted acetophenones and methyl benzoates: steric hindrance and inhibited conjugation. *Magn. Reson. Chem.* **2004**, 42, 844-851.

8. Akhtar, W. M.; Armstrong, R. J.; Frost, J. R.; Stevenson, N. G.; Donohoe, T. J. Stereoselective Synthesis of Cyclohexanes via an Iridium Catalyzed (5 + 1) Annulation Strategy. *J. Am. Chem. Soc.* **2018**, 140, 11916-11920.

9. Labes, R.; Battilocchio, C.; Mateos, C.; Cumming, G. R.; Frutos, O. de; Rincón, J. A.; Binder, K.; Ley, S. V. Chemoselective Continuous Ru-Catalyzed Hydrogen-Transfer Oppenauer-Type Oxidation of Secondary Alcohols. *Org. Pocess Res. Dev.* **2017**, 21, 1419-1422.

10. Rao, X.; Ishitani, H.; Yoo, W.-J.; Kobayashi, S. Zirconium-β Zeolite-Catalyzed Continuous-Flow Friedel-Crafts Acylation Reaction. *Asian J. Org. Chem.* **2019**, 8, 316-319.

11. Meng, G.; Szostak, M. Palladium-catalyzed Suzuki-Miyaura coupling of amides by carbon-nitrogen cleavage: general strategy for amide N-C bond activation. *Org. Biomol. Chem.* **2016**, 14, 5690-5707.

12. Xu, F.; Li, D.; Han, W. Transition-metal-free carbonylation of aryl halides with arylboronic acids by utilizing stoichiometric $CHCl_3$ as the carbon monoxide-precursor. *Green Chem.* **2019**, 21, 2911-2915.
